# Establishing a national research software award

**DOI:** 10.12688/openreseurope.16069.1

**Published:** 2023-10-25

**Authors:** Isabelle Blanc Catala, Roberto Di Cosmo, Mathieu Giraud, Daniel Le Berre, Violaine Louvet, Sophie Renaudin, College of experts for source code and software Committee for Open Science

**Affiliations:** 1 Ministère de l’Enseignement supérieur et de la Recherche, F-75000 Paris, France; 2 Inria, Université Paris Cité, F-75000 Paris, France; 3 Univ. Lille, CNRS, Centrale Lille, UMR 9189 CRIStAL, Centre de Recherche en Informatique Signal et Automatique de Lille, F-59000 Lille, France; 4 Univ. Artois, CNRS, UMR 8188, Centre de Recherche en Informatique de Lens, F-62300 Lens, France; 5 Univ. Grenoble Alpes, Grenoble INP Institute of Engineering, CNRS, UMR 5224 LJK, Laboratoire Jean Kuntzman, F-38000 Grenoble, France; 6 Direction de la recherche clinique et de l’innovation, Assistance Publique - Hôpitaux de Paris, F-75000 Paris, France

**Keywords:** open science, research software, open-source software, software engineering, research policy, institutional support, research infrastructure, research assessment, knowledge sharing

## Abstract

Software development has become an integral part of the scholarly ecosystem, spanning all fields and disciplines. To support the sharing and creation of knowledge in line with open science principles, and particularly to enable the reproducibility of research results, it is crucial to make the source code of research software available, allowing for modification, reuse, and distribution.

Recognizing the significance of open-source software contributions in academia, the second French Plan for Open Science, announced by the Minister of Higher Education and Research in 2021, introduced a National Award to promote open-source research software. This award serves multiple objectives: firstly, to highlight the software projects and teams that have devoted time and effort to develop outstanding research software, sometimes for decades, and often with little recognition; secondly, to draw attention to the importance of software as a valuable research output and to inspire new generations of researchers to follow and learn from these examples.

We present here an in-depth analysis of the design and implementation of this unique initiative. As a national award established explicitly to foster Open Science practices by the French Minister of Research, it faced the intricate challenge of fairly evaluating open research software across all fields, striving for inclusivity across domains, applications, and participants. We provide a comprehensive report on the results of the first edition, which received 129 high-quality submissions. Additionally, we emphasize the impact of this initiative on the open science landscape, promoting software as a valuable research outcome, on par with publications.

## Plain language summary

Software is crucial for modern research. For the goals of open science, reproducibility, and wider reuse, sharing software source code and acknowledging software development are essential. In France, in 2021, the Minister of Higher Education and Research introduced the National Plan for Open Science.

The plan highlights the role of open-source software in academia and aims to give software the same recognition as publications and data. A part of the plan is the introduction of a National Award to recognize open-source research software contributions. This award acknowledges software projects and their teams, which have often worked without much recognition. It also emphasizes the importance of software as a research output, hoping to inspire future researchers.

This article examines the award’s design and implementation. It addresses the challenges of assessing open research software from different research fields. In the first edition of the award, there were 129 high-quality submissions, indicating the award’s potential to shift perspectives on software’s role in open science, aligning it with the importance of academic publications. Through a detailed account of our experiences and the insights gained, we aim to provide a reference for other countries or institutions considering to establish similar recognitions.

## Introduction

**Figure 1.**
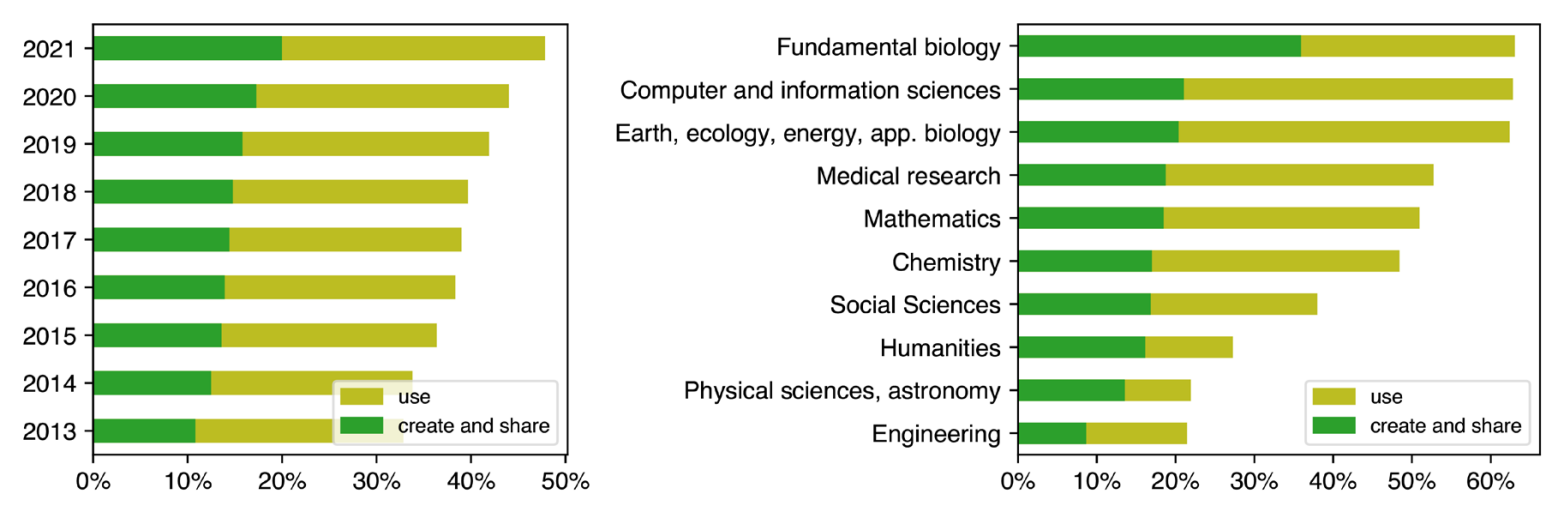
Software use and creation/sharing mentioned in French research articles by year (left), and, in 2022, by domain (right). Data from 2.

The development and diffusion of software has become a crucial part of the scholarly ecosystem ^1^, and the latest version of the French Open Science Monitor ^2^ now provides compelling evidence that software using and sharing span across all fields and disciplines ( Figure 1).

Software is not merely a tool, but a form of *executable knowledge* that is written by humans for humans, in the form of software source code, and later turned into *executable code* for machines. The importance of software in research cannot be overstated: it enables scientists to perform complex simulations, data analysis and modeling. The precision of software source code in implementing and describing data generation and collection, analysis, visualization, transformation, and processing cannot be matched by scholarly articles alone. As stated in the French policy on data, algorithms and open source ^3^ and the Second National French Plan for Open Science (PNSO2) ^4^, “software plays a key role in scientific research, as it is both a tool, a result, and a subject of study. Making software source codes available, with the ability to modify, reuse, and distribute them, is a major challenge to allow the reproducibility of scientific results and to support the sharing and creation of knowledge in an open science approach.”

To address this challenge, proper mechanisms need to be put in place to ensure the preservation and referencing of software source code ^5, 6^, as well as build awareness about the complexity of supporting reproducibility ^7, 8^, and provide proper training for researchers and engineers. In recent years, with growing awareness of the relevance of software in research, we have seen a variety of initiatives focused on software in academia: translations for software of basic principle designed for data description like FAIR ^9, 10^ or data citation ^11^; publications of software descriptions ^12, 13^, or reproducible research ^7, 14^; indexing software relevant to specific disciplines ^15, 16^.

In parallel with these efforts, it is of paramount importance to promote software development as a valuable research activity, and research software as a key enabler for Open Science/Open Research, recognizing in the careers of academics their contributions to high quality software development. This was stated in the November 2018 Paris Call on Software Source Code ^17^, and clearly put forward in the agreement on reforming research assessment published on July 20, 2022 ^18^. This is still a challenge today ^1^: Promoting software development is not an easy task, due to the many kinds of software projects and the broad varieties of expertise involved ^19^. Designing, coding, debugging, maintaining, documenting, testing, support, and management, are activities that require *specific skills*, ranging from scientific and technical knowledge, to community building and management, to writing and maintaining documentation that facilitates the use and appropriation of software.

### An award to advance recognition of research software

For all these reasons, a proper integration of software contributions in the evaluation of the research outcomes of individuals and institutions will take time, but it is important to start the process. As one of the means to advance the recognition of software as a research outcome on par with publications, the French Ministry of Research decided in 2021 to establish a national Open Science award for research software. Awarded since 2022, the *Prix Science Ouverte du Logiciel Libre de la Recherche* draws the scientific community’s attention to exceptional achievements as well as to the most promising ones, giving visibility to software that can serve as a model for future generations of scientists ^2^.

This initiative is different, in many respects, from other software awards that were established a long time ago in specific research fields, some of the most prestigious ones being the ACM Software Systems award ^20^, the ACM Programming Language Award in Computer Science and the Gordon Bell prize ^3^ or dedicated software competitions ^21^.

Focused explicitly on research software, the award we present here is set in the framework of Open Science, thus it only considers software that is distributed as open source. Being cross-disciplinary, it explicitly recognizes the many facets of software contributions that go beyond the technical and scientific ones ^4^. Sponsored by the Ministry of Higher Education and Research in France, its scope is restricted to software created or improved at the national level.

As such, it comes with specific challenges, and this article provides an in-depth view of the way this award has been designed and set up, a report on the results of the first edition in 2022 and the updates for the 2023 edition, as well as the lessons learned from the intense work that involved a significant number of experts from a variety of domains. We believe that this will provide a solid basis for establishing other national or international awards for research software. In the following, we discuss the institutional promotion of open research software, we present the steps taken in setting up this national prize, especially to ensure diversity in the awarded projects, and we report on the first edition of the prize.

## Establishing a national award

The National Open Science Research Software Award can act as a positive incentive for research institutions to promote and support software development and dissemination that benefit a wide scientific audience. From a research institution perspective, a national award is relevant for a plurality of objectives:

- *Identification of software production.* Research software has traditionally been difficult to track for research administration, and currently, limited resources are dedicated to software management, especially outside the software engineering or computer science communities. The call for applications for the award is an opportunity for the administration to engage with their research teams and learn about the software being developed across their organizations.
- *Opportunity for technology transfer.* A software award can foster collaboration within academia and partnerships with developer communities, and non academic actors locally or internationally.
- *Recognition and visibility of faculty.* Awards can have a significant impact on the visibility of authors and teams, leading to increased collaborations, but also to increased citations and recognition of their expertise.
- *Visibility of the institution.* Highlighting the achievements of research teams can raise the institution’s profile in the scientific community, contributing to talent retention and attraction. It can also be used as leverage to attract more funding for software projects.

### Defining research software

A key preliminary step in this endeavour is to define precisely which software falls in the scope of the award. As a first step, we focus on *research software*. This is software designed, maintained, and/or used by scientists and/or research institutions. It is developed to meet a specific need of science, hence it results from research work and/or enables scientific work, which is notably valued by publications before/on/around/with the software. We acknowledge the broad variety of forms that software can take: standalone and/or in interaction within an ecosystem, a platform, middleware, or a library, module or plugin of another software. Furthermore, to be eligible for an Open Science award, research software should be licensed as *free and open source software*, typically under one of the licenses approved by the Open Source Initiative ^5^. The “gray zone” for the status of some projects will be discussed below.

## Challenges and solutions in establishing the award

Establishing a *national* prize designed to take into account the *diversity* of software contributions *aligned with the Open Science vision* and *across all disciplines* turned out to be a far from trivial task. In this section, we report the main challenges and the solutions that have been retained to address them.

**Delimiting the national scope.** Supported by the French Ministry of Higher Education and Research, the prize is intended to be *national*. It aims to highlight research software which has been supported by a French university or research institute. In practice, eligibility had to be assessed using several criteria. To be eligible, a software should have been initiated or should have received a strong contribution to its development from a French institution. As such, software maintained by an international community is eligible provided it can demonstrate that kind of support. In particular, it was decided that a research software developed during a PhD or postdoc in France can be submitted by its author even if they no longer are affiliated to a French institution. The submission itself should be endorsed by one of the active developers of the software. This scope had a significant influence on other topics, particularly on domain diversity, as discussed below.

**Taking into account software diversity: Development practices.** Research software is often developed by researchers who are *not* professional software engineers. Even researchers in computer science may specialize in theoretical or modeling aspects rather than software development. While software engineering domains are expected to apply state-of-the-art practices, other domains may not follow these practices. For example, the Covid-sim C++ source code was deemed sound and correct as a simulator despite not being received well by software engineers ^26^. Non-professional software developers may also become expert developers in their research area.

To take this diversity of practices into account, it is crucial to refrain from looking at research software only through the software engineering lens. Promoting good software engineering practices is important as they are necessary skills for producing good quality code, but this must not be a hard requirement for the award.

**Taking into account software diversity: Maturity.** Some research software have been developed for decades, while others are very new, for example a prototype written during a PhD thesis. Some software have institutional support and significant resources for their development, others are supported by a large international community, while the remaining ones are maitained by a few, or just one, individuals without any particular help. A direct comparison of the research software with the above criteria often favor established projects with institutional support, but the committee aimed to consider the challenges and achievements of smaller and more recent projects as well. As such, the achievements of a software was related to the resources available.

As will appear in the Results section, the vast majority of the submissions in 2022 corresponded to software started between 2005 and 2019 (96 out of 129). Nevertheless, 46 submissions were recent (started after 2015), which corresponds to the rise of Open Science. However, it was quite difficult to compare those young software against mature and well-established ones, especially in the first edition of the prize: submissions of mature software denoted software that "survived" more than a decade of research (often two decades, three decades for the oldest). Those software have built a strong community, opened dedicated research directions which made them *de facto* eligible in all categories. As such, in 2023, the jury also has the possibility to award in each category an *emerging software* to recognize promising recent software (less than five years old).

**Dealing with the open-source gray area.** To be in accordance with the Open Science vision, research software should be licensed as *free and open source software* to be eligible. This property should be easy to check, by just looking at the licence. In practice, there are more complex situations. Some significant research software have started to be developed before the free and open source licenses were published, and never added a license afterwards since it would require much effort to contact all contributors to do so. Some other projects, despite being more recent, are made available without a license. The submission process, which requires selecting an open source license, incites the software development team to take into account that important aspect. It may be the case that the software itself is open-source, but requires a proprietary platform to be executed (think *e.g.* of Matlab scripts), or that some parts of the software may not be open-source ( *e.g.* in an industrial context). Generally speaking, while it is essential that the scientific output should be as open as possible, it was decided to have the largest definition as possible of openness, *i.e.* even within closed ecosystems, to promote reproducibility and enable others to build upon such research.

**Awarding community.** Software is often not developed by a single person, and the prize wishes to also recognize the role of the community behind the software. The prize is thus not awarded to individuals but to the software and the research effort itself. It is worth noting that individuals with short-term contracts such as PhD students, post-doctoral fellows, and engineers often make key contributions to research software, and may even build them from scratch. They may have left their research institution or even a research career when the software is mature or well-recognized enough to win the prize. We believe that in all cases, the fact that the software they contributed to is distinguished will help them for their career.

**Defining the award categories.** The broad spectrum of research software led us to consider initially several other categories, such as “societal and economic impact”, but it was finally decided to keep the framework simple, with only three categories: *scientific/technical*, *documentation*, and *community*.

These categories are very broad and are intended to highlight different aspects of research software. The first one aims to showcase successful research results implemented in software or obtained with software. The second one focuses on the importance of the documentation of the software, both from a user and a developer perspectives. The last one highlights the ability to build and sustain a community of developers, maintainers, and/or users around the software.

A special Jury Prize allows the jury to distinguish remarkable research software from a different perspective than the previous categories. Starting in 2023, submissions may specify one or several award categories, but the committee may reassign some software to other categories.

**Composition of the committee.** Considering all of the above, it is essential to convene a committee that is not only legitimate but also has a broad spectrum of expertise and sensibility. To achieve this goal, the committee ^6^ included members from various research areas, both from academia and industry, as well as from the open-source community. Some of these members had extensive knowledge of research software in their respective domains, which was essential to contextualize the submissions.

**Evaluation criteria.** The criteria considered by the committee apply to the application itself. This includes clarity, writing quality, completeness, number of developers, relevance for research, and publications around it, assessment of academic, societal, and industrial impact, number of software citations, community organization, and user and developer documentation. Furthermore, the criteria also apply to public information obtained directly from the project’s website or code repository, such as the activity of the source code repository, contributors, issue tracking, and forks. Although not all submissions used a source code repository, this information was helpful to break ties among comparable projects.

The concept of community was also discussed. Some software may have a pure research community, while others may have an impact on the socio-economic world, with varied contributions (code, extensions, tutorials, books, consortiums, organizations). The organization of events (workshops, conferences, meetings) to animate the user or contributor communities has also been taken into account. It is worth noting that research software may require very specific knowledge to understand its inner details, which can limit the number of people who can effectively contribute to the code. However, this does not prevent them from being widely used and building a dedicated (and sometimes large) user community.

**Open science awards ceremony.** The main goal of the prize is to recognize and showcase the exceptional quality of the teams behind the research software, and not just to provide a monetary reward, which can be negligible compared to the positive impact that the award may have on the project’s ability to secure additional funding. During a public ceremony, the Minister of National Education and Research awarded a trophy to each project laureate (see Figure 3).

## Results and discussion

The first edition of the National Open Science Research Software Award was held in 2022, with 129 complete submissions received. The submissions were of a very high quality, demonstrating the impact that high-quality research software can have. Since this was the first edition of the prize, the jury had some freedom to develop the award selection process, that is now better formalized for the 2023 edition.

### A diversity of submissions

**Figure 2.**
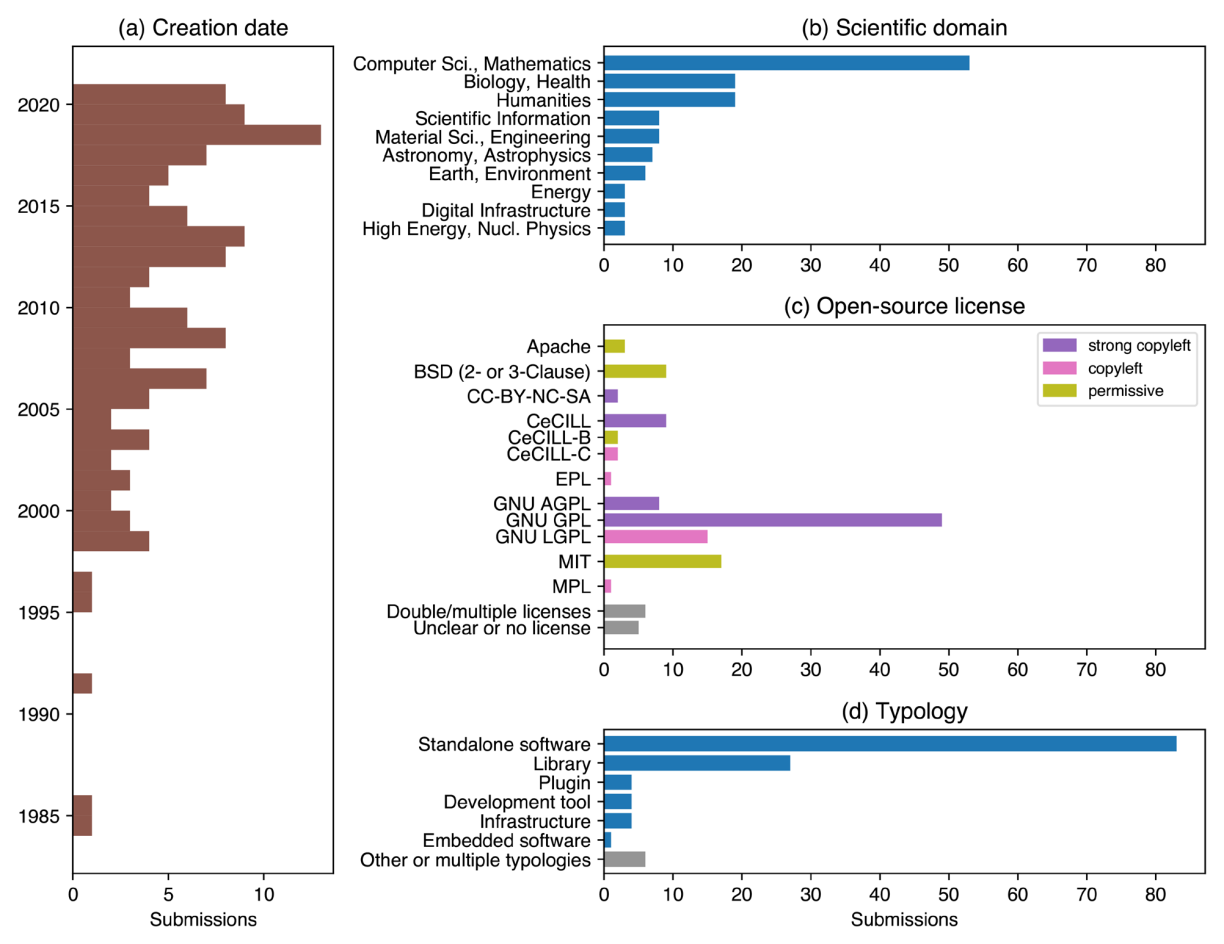
The 129 submissions to the first edition of the “Prix Science Ouverte du Logiciel Libre de la Recherche”, in 2022, show a variety of maturity, domains, licenses, and software typologies. Note that, in 2022, the scientific domains of the submissions was not given by the applicants but inferred from the submissions.

Very mature software were submitted, among which some initiated in the 80’s, while many submissions were much more recent (2018–2021, Figure 2a). While many applications come unsurprisingly from computer science and mathematics, the remaining ones come from a wide variety of domains, including humanities ( Figure 2b, see end of section).

**Figure 3.**
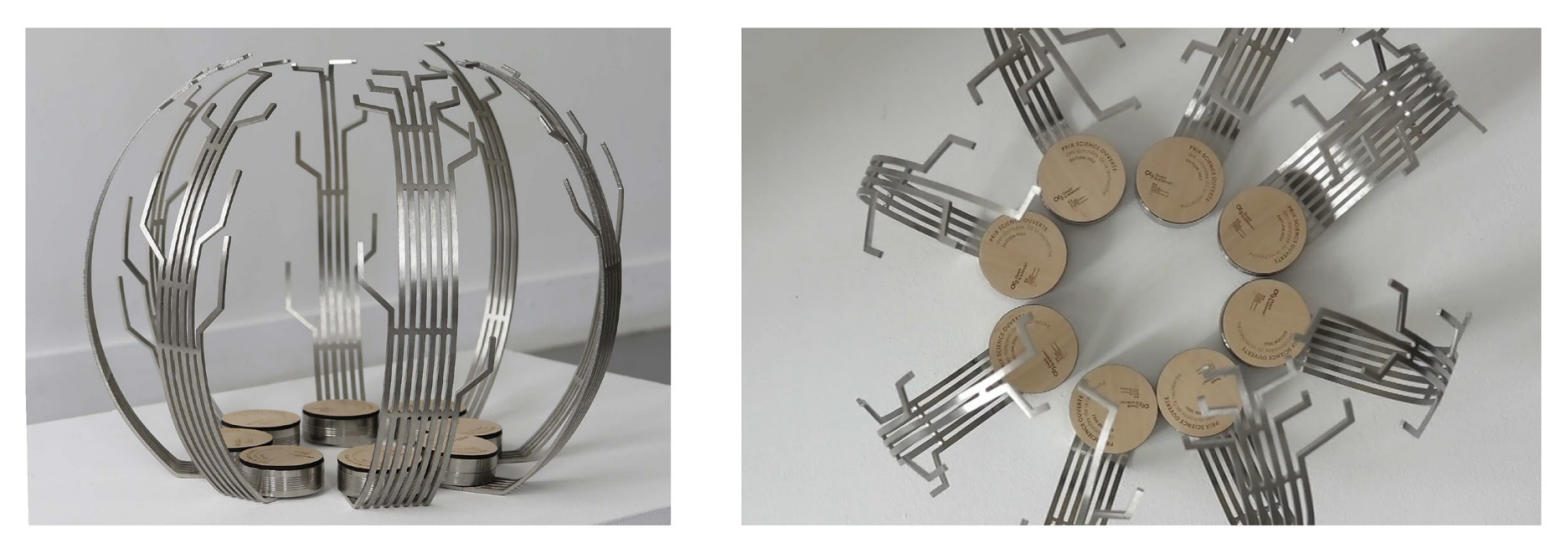
The award trophies were designed by Alix Nadeau, Rose Vidal, Hugo Bijaoui, and Lorris Sahli, who are students at the École des Arts décoratifs (Paris). The trophies symbolize how collaboration through open code and data can improve science outreach. Each trophy has a unique shape, and the designs and code used to produce them are open-licensed ( https://github.com/LorrisEnsad/Trophee_OpenScience), reflecting the principles of open science that the award seeks to promote.

The vast majority (94%) of submissions used OSI-approved licenses ( Figure 2c). They include the GNU GPL (49 submissions, including 35 in GPLv3/GPLv3+), LGPL, and MIT, as well as the French CeCILL licenses, particularly the CeCILL(-A) variant, which is compatible with the GNU GPL.

The nature of the software itself has implications in the way it had to be evaluated ( Figure 2c). For instance, libraries are often more easily reusable than standalone software. Additionally, there is a greater chance of having contributors for a library since it is primarily aimed at developers. This is also generally true with software for a computer science audience compared to software used in other scientific domains. Libraries also generally benefit from standardized documentation and distribution channels depending of the language used, which can also favor their adoption. Another important aspect is the target audience of the software. Some are specifically addressed to their scientific community; others make some results accessible in other research areas or outside academia. Those different audiences lead to different documentation requirements and community management. It should also be noted that some of the submitted software were designed to support data collection and manipulation, rather than results production. Both are valuable. However, for the purpose of this award, the jury focused solely on software that contributed to results production.

### Award process

In the initial screening, each submission was reviewed by three members of the committee. Each member provided a feedback on each submission: *strong support*, *support*, *do not support*. All submissions that received at least two *strong support* were considered eligible to an award. Those 40 submissions were then reviewed by all other members of the committee. The submissions were classified by scientific domain in comparable software sets. The most promising software in each set were selected, which ended up in 15 very different, not easily comparable software.

The final ranking was obtained from that selection of 15 software, by awarding a software in each category (4), and providing an honorable mention to some remaining remarkable software in each category (6). With 10 laureates, the committee believes they reached the goal of awarding software from diverse scientific domains, diverse institutions, with diverse practices. These 2022 awards cover a variety of fields, including fondamental topics such as proof assistance ^7^, machine learning ^8^, multi-agent systems ^9^, as well as more applied topics such as routing in computer architecture ^10^, computer music ^11^, astrophysics ^12^, bioinformatics ^13^, brain-computer interfaces ^14^, seismology and volcanology ^15^, and linguistics ^16^.

## Conclusion and perspectives

In the past, several awards have been established to recognize remarkable software projects within specific disciplines, communities, or organizations. However, the aim of a National Open Science Research Software Award is distinct: it serves to acknowledge and incentivize the development of high-quality research software across all disciplines, and to establish software development as a research output on par with publication. The creation of the "Prix Science Ouverte du Logiciel Libre de la Recherche" posed challenges due to the diverse scientific domains and practices involved in conceiving, developing, documenting, and distributing research software while also fostering a community. The submissions and awardees of the first edition demonstrated the variety of software that aligns with the Open Science vision.

In France, this award is endorsed by the Ministry of Higher Education and Research, which organizes it annually following the seminal 2022 edition. Promoting open-source software at the institutional level will enhance the reproducibility of scientific results and support the sharing and creation of knowledge within an open science framework. We are delighted to see that an award has already been announced in Australia in 2023 ^17^. We now encourage institutional stakeholders worldwide to replicate this initiative, freely building on the model and experience described here.

## Data Availability

Recherche Data Gouv Dataverse: Données anonymes concernant les candidatures au premier prix science ouverte du logiciel libre de la recherche, https://doi.org/10.57745/HN6O85 For each of the 129 submissions, this dataset includes metadata on the start date of development, programming language(s), license(s), and scientific domain. Personal information was removed. Data are available under the terms of the Etalab Open License 2.0.

## Footnotes

^1^ While in some disciplines and some countries the contributions of software developers are taken into consideration by institutional authorities ^19^, other countries ignore completely software development in the evaluation process for researchers.

^2^ https://www.ouvrirlascience.fr/

^3^ There are also a few other awards specifically for Free Software ^22^ or Open Source ^23^, for specific institutions ^24^, or specific fields in a discipline ^25^.

^4^ Sometimes, a software system may have high scientific value but lack strong software engineering skills, as was put on the limelight with a simulation software for the COVID-19 pandemic ( link).

^5^ The OSI-approved licenses are available at https://opensource.org/licenses/

^6^ The committee’s composition is available at https://data.enseignementsup-recherche.gouv.fr/explore/dataset/ fr-esr-jury-prix-science-ouverte/table/

^7^ https://coq.inria.fr

^8^ https://scikit-learn.org

^9^ https://gama-platform.org

^10^ http://coriolis.lip6.fr

^11^ https://faust.grame.fr

^12^ https://gammapy.org

^13^ https://vidjil.org

^14^ http://openvibe.inria.fr

^15^ https://ipgp.github.io/webobs

^16^ https://sppas.org

^17^ https://ardc.edu.au/article/enter-ardc-eureka-prize-for-research-software/
