# Supplementary material for: Establishing a national research software award
Source: Open Res Eur. 2023 Oct 25;3:185. [Version 1] doi: 10.12688/openreseurope.16069.1 (PMC10674088; doi:10.12688/openreseurope.16069.1)
Supplement: Peer review history

# Reviewer report: Reviewer response for version 1

**DOI:** 10.21956/openreseurope.17349.r35882

**Published:** 2023-11-24

**Authors:** Jesús M. González Barahona

**Affiliations:** 1 Universidad Rey Juan Carlos, Móstoles, Community of Madrid, Spain

The article presents the experience of running the French National Award to promote open-source research software. It discusses its rationale, in the context of the recognition of the importance of free, open source software (FOSS) for open science, the details of how it was targeted, and the software that applied for it evaluated, and a brief analysis of the projects presented to the first edition of it.

 I really enjoined reading the article. I find the initiative very interesting in the context of the recognition and promotion of open science practices, and the fact it is described in such detail helps a lot to understand it, and to reproduce it in other contexts.

 The rationale for the described initiative is well described, with good detail, and put in context appropriately.

 The article is not much about opinions, but mostly about facts, See below, in "other comments", for some aspects that could be discussed in some more detail, but in general I see only a few opinions, and when presented, they are adequately put in the context of the facts that raised them.

 To my knowledge, factual statements are correct and supported. In this case, they are usually not supported by citations, but by facts (related to the design and execution of the process for granting the awards).

 The article is well written and easy to read, and the language is accessible.

 The article does not discuss in depth next steps and recommendations. Given the uniqueness of the initiative described, and its importance, this is the only drawback that I find in the article. I encourage authors to describe the lessons they learned (some of them are mentioned briefly as changes for the 2023 edition), but also how do they think the award could evolve in the future, how the initiative could be transplanted to other realms (other countries, or international awards of the same kind, for example), and in general, next steps that they may recommend, after their experience with the award, in the field of recognizing the importance of FOSS in open science.

 All in all, I find the article interesting, relevant and sound.

 Some other comments. Most of them relate more to issues that I personally find interesting to discuss more than anything else. However, given the unique opportunity that authors had in considering these issues, having more details on these aspects could also be important for many other readers of the article.

 * Abstract & plain language summary: "129 high-quality submissions". If this is the total number of submissions, maybe it is better to just write "129 submissions". Even if all of them were good, it is difficult to assess that all were high-quality except if that is backed by some fact (and likely, there is no need for the qualification at this point).

 * Establishing a national award: "Recognition and visibility of faculty." Of course, I don't know if that was one of the objectives, but beyond recognition, I see opportunities for facilitating the evaluation of activities related to the production of research software as a research activity, which could be important for a more fair evaluation of research activities in the context of open science.

 * Defining research software: "This is software designed, maintained, and/or used by scientists and/or research institutions. It is developed to meet a specific need of science,..." Maybe it would be interesting to have a more nuanced description of the definition. My guess is that the authors are considering software that is described by both sentences simultaneously, not either of them, But it is not clear from the text. If my guess is right, I would suggest a more explicit definition such as "Research software was defined as software fulfilling the following conditions: it is designed, maintained, and/or used by scientists and/or research institutions; it is developed to meet a specific need of science,..." In fact, since in different context "research software" is defined in different ways, this definition, if clear and detailed enough, could be a "canonical" definition, suitable for being used by others.

 * Delimiting the national scope. Being FOSS so prone to international collaboration (specially if it succeeds), I may think that this condition could cause some problems in practice, and could for example make it difficult to award the work of French developers heavily involved in medium-large international FOSS projects, even if their contributions were similar or more important (in "absolute" value) than other relatively smaller contributions to software supported o originated by French institutions. Some discussions about the rationale of this delimitation (which seems mostly based on "institutional support" as opposed to "individual contribution") would be rather interesting, since similar problems could be found in other situations, and in any case, from a FOSS perspective, considering institutions above persons (if that were the case for this matter) could deserve an explanation. However, I understand that all of this likely arises from the problems of defining a national scope (which was needed due to the funding of the award), to many cases which are international by nature.

 In fact, this matter could be a part of a deeper issue, which I don't find discussed in the article: the award seems to be targeted at "software", not at "people producing software". Even when that is of course a sensible decision (to visualize and recognize software instead of individual or group contributions), since both approaches could have been reasonable, the reasons for the specific approach chosen could be worth some discussion in the article. I see this commented later in "Awarding community", "The prize is thus not awarded to individuals but to the software and the research effort itself." But this seems contradictory with the eligibility criteria, which, as I said, seems to be institution-driven.

 * Dealing with the open source gray area. "it was decided to have the largest definition as possible of openness, i.e. even within closed ecosystems, to promote reproducibility and enable others to build upon such research." Nothing is said in this sentence about the software that lacked a license (which is described in the previous sentences). It would be interesting to know if such software was also considered, if it was required to decide on a license before being considered, and if not, how it was solved the problem that software without a license, can hardly be used and redistributed without some legal risk.

 * Evaluation criteria. It is mentioned the importance of the community, and the distinction between development and user communities. It would be interesting to know how the importance of those communities was weighted, if there were some specific criteria for that. Since they really represent very different aspects of a software project, but are also related (for example, people in the user community may become people in the development community at some point).

 If the jury used some kind of template for scoring, detailed criteria, or whatever, that would be a nice complement to the article, maybe as an annex, and would help for the reproducibility of the experience elsewhere.

 * A diversity of submissions (and some other sections). The description of the projects seem to correspond only to the 2022 edition. Some parts of the article suggest that is the case, but since the 2023 edition is also mentioned, it would be worth making it clear.

 * A diversity of submissions. "For instance, libraries are often more easily reusable than standalone software" I guess authors have in mind "reuse of the software as a component, in other software". However, "reuse" can also mean "reuse n some other research team than in the original team building the software". It could be interesting to complement this sentence explaining that the second kind of reuse, which is core in open science, is not more difficult to evaluate for standalone software (maybe it is easier, since the use of the software in those cases is more evident).

Where applicable, are recommendations and next steps explained clearly for others to follow? (Please consider whether others in the research community would be able to implement guidelines or recommendations and/or constructively engage in the debate)

Partly

Does the article adequately reference differing views and opinions?

Yes

Are all factual statements correct, and are statements and arguments made adequately supported by citations?

Yes

Is the rationale for the Open Letter provided in sufficient detail? (Please consider whether existing challenges in the field are outlined clearly and whether the purpose of the letter is explained)

Yes

Is the Open Letter written in accessible language? (Please consider whether all subject-specific terms, concepts and abbreviations are explained)

Yes

Reviewer Expertise:

Analysis of software development, free open source software, software analytics, XR data visualization

I confirm that I have read this submission and believe that I have an appropriate level of expertise to confirm that it is of an acceptable scientific standard.

# Reviewer report: Reviewer response for version 1

**DOI:** 10.21956/openreseurope.17349.r35884

**Published:** 2023-11-21

**Authors:** Karthik Ram

**Affiliations:** 1 University of California Berkeley, Berkeley, California, USA

**Review of Establishing a national research software award**

**Summary**:

 "Establishing a National Research Software Award," authored by Isabelle Blanc Catala *et al *explores the critical role of software in academic research. The paper discusses the inception of a National Award for open-source research software in France, introduced as part of the French Plan for Open Science in 2021. This initiative seeks to acknowledge and promote exceptional research software projects and their teams, many of whom have made substantial contributions over the years with minimal recognition. Additionally, the award aims to highlight software as a key research output and to inspire future researchers in this field.

 The article effectively outlines the establishment of this award, underscoring its significance.

**Comments**:

 The abstract, plain language summary and introduction strongly emphasize the necessity of open-source software for reproducibility and broader reuse. While advocating for open-source research software, especially those funded publicly, is commendable, it is not a strict prerequisite for reproducibility. Merely providing binaries under an open license could suffice for reproducing and validating previous results. Therefore, I suggest that you refine this argument, underscoring that open-source software not only facilitates the examination of code for accuracy and building upon existing work but also supports reproducibility and reuse. For instance, open-source software, being freely available and modifiable, endures even if the original creators cease maintenance. The paper later adopts a more flexible stance on openness (bottom of page 6), so maintaining this perspective throughout would be consistent.

 While the paper effectively narrates the importance of software in research and the rationale behind instituting a national award, it is somewhat lacking in detailing the review criteria and process. The Evaluation Criteria section (page 6) could benefit from more explicit information. For example, clarifying the metrics for assessing a software's community impact would add value. Is it possible to provide additional details about the evaluation rubric, or would that exceed the scope of this article?

 An important aspect that seems overlooked is how the award's criteria might secondarily elevate the quality of software in France. Clear criteria for the award could incentivize software projects to adopt best practices, thereby improving their standards even if they don't win the award (e.g., "software projects made available without a license"). The award's existence alone could thus serve as a model for exemplary practices within the French research community.

 The discussion could potentially expand on how the award might evolve based on learnings from this iteration. Are there any considerations regarding the future trajectory of the award, especially in light of the varying number of submissions across different fields?

**Minor Suggestions:**

 Consider rephrasing the sentence: "The development and diffusion of software has become a crucial part of the scholarly ecosystem." A more straightforward and clear alternative could be: "The widespread development and adoption of software is increasingly integral to the scholarly ecosystem." This revision enhances clarity and simplicity.

Where applicable, are recommendations and next steps explained clearly for others to follow? (Please consider whether others in the research community would be able to implement guidelines or recommendations and/or constructively engage in the debate)

Yes

Does the article adequately reference differing views and opinions?

Yes

Are all factual statements correct, and are statements and arguments made adequately supported by citations?

Yes

Is the rationale for the Open Letter provided in sufficient detail? (Please consider whether existing challenges in the field are outlined clearly and whether the purpose of the letter is explained)

Yes

Is the Open Letter written in accessible language? (Please consider whether all subject-specific terms, concepts and abbreviations are explained)

Yes

Reviewer Expertise:

Open source, open science, reproducible research, computational biology, global change biology, software sustainability

I confirm that I have read this submission and believe that I have an appropriate level of expertise to confirm that it is of an acceptable scientific standard.
